# Embryogenesis in the parasitic nematode *Heterodera glycines* is independent of host-derived hatching stimulation

**DOI:** 10.1186/s12861-016-0144-7

**Published:** 2017-01-11

**Authors:** Sita Thapa, Jayna A. Patel, Ursula Reuter-Carlson, Nathan E. Schroeder

**Affiliations:** Department of Crop Sciences, University of Illinois at Urbana-Champaign, 1102 S. Goodwin Ave, Urbana, 61801 IL USA

**Keywords:** Soybean cyst nematode, SCN, Root knot nematode, Cleavage, Post-embryonic development, Ventral nerve cord

## Abstract

**Background:**

Many parasites regulate their development to synchronize their life cycle with a compatible host. The parasitic nematode *Heterodera glycines* displays incomplete host-mediated hatching behavior wherein some *H. glycines* individuals hatch only in the presence of a host-derived cue while others hatch in water alone. Furthermore, *H. glycines* shows variable hatching behavior based on oviposition location. The mechanisms regulating this hatching variability are unknown. In this study, we established a detailed timeline of the *H. glycines* pre-hatch development from early embryogenesis to the pre-hatched J2. These descriptive data were then used to test hypotheses regarding the effect of host stimulus and oviposition location on pre-hatch development.

**Results:**

We found that *H. glycines* develops from a single-cell egg to a fully formed J2 in approximately 172 hours. The stylet-based mouthpart, which is used to pierce the eggshell during hatching, is not completely formed until late in pre-hatch J2 development and is preceded by the formation of stylet protractor muscles. We also found that the primary motor nervous system of *H. glycines* did not complete development until late in pre-hatch J2 development. These data suggest possible structural requirements for *H. glycines* hatching. As expected, exposure of *H. glycines* eggs to host-derived cues increased the percentage of nematodes that hatched. However, exposure to hatching cues did not affect pre-hatch development. Similarly, we found no obvious differences in the pre-hatch developmental timeline between eggs laid in an egg sac or retained within the mother.

**Conclusions:**

The pattern of early embryonic development in *H. glycines* was very similar to that recently described in the related parasitic nematode *Meloidogyne incognita*. However, the speed of *H. glycines* pre-hatch development was approximately three times faster than reported for *M. incognita*. Our results suggest that hatching stimulants do not affect embryogenesis itself but only influence the hatching decision once J2 development is complete. Similarly, the oviposition location does not alter the rate of embryogenesis. These results provide insight into the primary survival mechanism for this important parasite.

## Background

The life cycle of most nematodes comprises embryogenesis followed by four juvenile stages (J1–J4) and the adult reproductive stage. Parasitic nematodes frequently include an infective stage in which development is arrested until a host is present and successfully infected [1]. In many species, this arrest occurs before hatching. In the absence of a host, the nematode remains within the relatively protected eggshell until it has received appropriate signals from the host. For example, eggs of the animal-parasitic nematode *Ascaris lumbricoides* do not hatch until after they have been ingested [2]. Most plant-parasitic nematodes hatch as J2s; following embryogenesis, they undergo the first molt within the egg. The egg of many plant-parasitic nematodes serves as the primary long-term survival stage. After leaving the protective environment of the eggshell, hatched J2s have limited energy reserves to find and infect a host [3]. To maximize the probability of success during this sensitive period, several species of plant-parasitic nematodes have evolved host-mediated hatching behaviors. Among plant-parasitic nematodes, the integration of host cues into the hatching process has progressed furthest in the cyst nematodes (*Globodera* spp., *Heterodera* spp.).

The hatching behavior of the parasitic nematode *Heterodera glycines* (commonly known as the soybean cyst nematode) is synchronized with its soybean host and, therefore, has a significant impact on control strategies [4]. *H. glycines* females produce several hundred eggs [5]. While some individual eggs will hatch in the absence of a host, the presence of soybean greatly increases the percentage of eggs that will hatch, thereby increasing the probability of emerging in the presence of a susceptible host. However, even in the presence of soybean and other conducive environmental conditions, many *H. glycines* individuals will remain in an arrested stage. This incomplete host-derived hatching stimulation is thought to serve as a hedge against a sudden deterioration in environmental conditions [6]. Complicating this behavior, approximately one third of *H. glycines* eggs are laid into a gelatinous sac (egg sac eggs), located at the posterior end of the female, while the remaining eggs are retained within the female body (encysted eggs). Upon death, the body of the dead female is referred to as a cyst, which serves as an additional layer of protection from the environment for the encysted eggs. There are differences in hatching behaviors between egg sac eggs and encysted eggs [7, 8]. The mechanisms regulating the complex hatching behavior of *H. glycines* are unclear.

To better understand the variability in hatching of *H. glycines*, it may be useful to have a more complete description of pre-hatch development. Compared to the well-studied nematode *C. elegans,* the development of most parasitic nematodes is not well-described. Previous research has found substantial variation in early embryonic cleavage patterns among nematode species [9, 10]. A previous description of the *H. glycines* life-cycle suggested that it took approximately six days for embyros to develop to J2s [11]. Separation of *H. glycines* eggs into embryos and juveniles can be accomplished using visual assessment via a dissecting microscope or through flow cytometry; this separation can then be used to increase developmental synchronization in hatching studies [8, 12, 13]. For example, Masler et al. directly compared hatching in *Meloidogyne incognita* with *H. glycines* following separation of eggs into embryos and juveniles [13]. However, a detailed description of the timing of early development in *H. glycines* is lacking and may be useful for understanding the hatching process. Recent work on the early embryonic development of the nematode *M. incognita* provides a comparative species within the primary plant-parasitic nematode clade to examine early cleavage events and a timeline from a single-celled embryo to hatching [14]. Here, we describe a similar detailed timeline of pre-hatch development for *H. glycines*.

The development of structures required for hatching in nematodes is not well described. Unlike *C. elegans*, *H. glycines* has evolved a protractible stylet mouth part that is used for both infection and opening of the eggshell during hatching. The development of the secreted cuticular-based stylet and its associated musculature is not understood. We recently demonstrated that hatched J2 *H. glycines* contain several additional neurons in the primary motor nervous system compared to *C. elegans* [15]. In this study, we examine the pre-hatch development of the stylet and ventral nerve cord (VNC) of *H. glycines*.

Since host cues greatly increase the hatching rate of *H. glycines*, we used our understanding of pre-hatch development to test the hypothesis that the presence of a host regulates the rate of *H. glycines* embryogenesis and that this could lead to differences in hatching rate. Several animal species use environmental cues to regulate developmental rates in order to achieve optimal hatching behaviors. For example, turtles adjust their rate of embryogenesis to establish synchronized hatching within a clutch [16, 17]. However, we found that host-derived cues and the location of egg deposition do not impact the rate of pre-hatch development in *H. glycines*.

## Methods

### H. glycines

*H. glycines* were grown on soybean (cv. Williams) in a 1:1 sand to soil mixture. Females were collected by mixing roots and soil with water and pouring over stacked 850 and 250-μm-pore sieves. Females were collected from the 250-μm-pore sieve and separated from root fragments and other debris by centrifugation in a 68% (^w^/_v_) sucrose solution [18]. The supernatant was poured again on 250-μm-pore sieves, washed, and recovered females were collected. For the pre-hatch development study, encysted and egg sac eggs were separated. Egg sacs were soaked for 1 minute in 0.5% NaOCl to dissolve the gelatinous matrix and poured over stacked 250 and 25-μm-pore sieves [8, 19]. Egg sac eggs were then collected from the 25-μm-pore sieve. To collect encysted eggs, we opened cysts manually. All eggs were obtained from three to four-week old females. Two-celled eggs were handpicked under a dissecting microscope and used within one day following extraction.

### Hatching stimulant preparation

Soybean root exudate was collected based on previous methods [4, 20]. Soybeans (cv. Williams) were grown in a 2:1 sand to soil mixture. After six weeks, the plants were removed from the soil mix, and the roots were rinsed with tap water to remove soil particles. The roots of four plants were incubated for 48 h in 400 ml of distilled water at 24 °C. The roots were excised at the hypocotyl, blot dried, and weighed. The concentration of host exudate was adjusted to 1.3 root-gram-hour as calculated by the formula $\frac{root\  mass\ (g) \times incubation\ (h)}{amount\ \mathrm{o}f\  root\  exudate\  obtained\ (ml)}$ [21]. Host exudate was sterilized by passing through a 0.2-μm cellulose acetate sterile syringe filter (VWR), and stored at−20 ° C.

### *H. glycines* hatching experiment

Hatching chambers consisted of a 30-μm nylon mesh (Genesee Scientific, CA) held between 30-mm (l)×38-mm (d) and 15-mm (l)×40-mm (d) plastic cylinders [22]. Hatching chambers were placed on the bottom of 5-cm Petri dishes (VWR). Approximately 1,000 eggs of mixed developmental stages were dispensed in hatching chambers with water or hatching stimulants. Hatching chambers were stored at 25 °C. Each treatment was randomly assigned and had three replications. Every 24 h, the hatching chambers were transferred to new Petri dishes and hatched J2s recovered from the Petri dish were counted. The experiment was repeated once. The data were analyzed in SAS with PROC MIXED for repeated measures with a compound symmetry covariance structure [23].

### *H. glycines* early embryo development and pre-hatch developmental timeline

Several methods were used to examine pre-hatch development. The pre-hatch development timeline was initially observed using a hanging drop method with modifications [24]. Single eggs were added to 6 μl of either water or a hatching stimulant in a concave well microscope slide and covered with a coverslip. We found that *H. glycines* embryos were very sensitive to pressure. Therefore, drops of petroleum jelly were placed at each corner of the coverslip to serve as spacers. Slides were kept in a humidity chamber at 25 °C between imaging. Development was observed using an inverted microscope (Zeiss Axiovert 200) with Differential Interference Contrast (DIC) microscopy at 400× magnification. A pre-hatch developmental timeline was created for both water and hatching stimulants based on at least 15 animals for each treatment. High-resolution images of major stages of the developing embryo were collected using an upright compound microscope with DIC optics at 1000× magnification. To further confirm our results with the inverted microscope and obtain higher resolution images, the early embryonic development of three animals from the fusion of the male and female pronuclei to the eight-celled stage was observed. Single eggs were placed on a 4% agarose pad. Coverslips were sealed with parafilm, rehydrated as needed, and incubated at 25 °C in a dark humidity chamber when not imaging. Images were taken every 30 minutes using an upright compound microscope with a mechanized stage (Zeiss M2 AxioImager and Zen software). Cell divisions were labeled based on *M. incognita* and *C. elegans* [14, 25].

### *H. glycines* post-embryonic stylet and VNC development

Two-celled eggs were incubated in water at 25 °C for 6–7 days to obtain pre-hatched J2s. Eggshells were gently opened in phosphate buffered saline with Triton X-100 (PBST: 8 g NaCl, 0.2 g KCl, 1.43 g Na_2_HPO_4_, 0.05% (^v^/_v_) Triton X-100 in 1 L) using pressure from fine forceps. These pre-hatch J2s were fixed in 2% formaldehyde at 4 °C overnight in 1-ml microcentrifuge tubes. The stylet protractor muscles were visualized by staining fixed nematodes with Alexa Fluor 488 phalloidin. Nematodes were transferred onto a 4% Noble agar pad with 20 μl of 5% (^v^/_v_) phalloidin. The nematode cuticle was punctured with a Micropoint laser to aid in the penetration of phalloidin. Slides were kept in a moist chamber overnight at room temperature and imaged the next morning. The VNC was visualized with 4’, 6-diamidino-2-phenylindole (DAPI) using a modification of previous methods [15]. Twenty early and late pre-hatched J2s were fixed in 2% formaldehyde, washed three times with PBST, and incubated in 50% methanol for 30 min. Nematodes were then washed three times with PBST and incubated in 0.2–0.5 μg/ml of DAPI overnight in the dark at room temperature. Twenty-eight hatched J2s were fixed in 4% formaldehyde and incubated in 100% methanol for at least four hours followed by overnight incubation in DAPI. Nematodes were stored at 4 °C before examination. Images were taken using a Zeiss M2 AxioImager with DIC and fluorescence. The VNC nuclei were identified based on their size and morphology [15, 26]. DAPI stained neurons appeared as highly condensed round fluorescent puncta [15, 26]. Neuron-like nuclei within the VNC were counted from immediately posterior of the retro-vesicular ganglion to immediately anterior of the pre-anal ganglion [15].

## Results

### *H. glycines* embryos develop much faster than *M. incognita*, but with a similar cleavage pattern

**Fig. 1:**
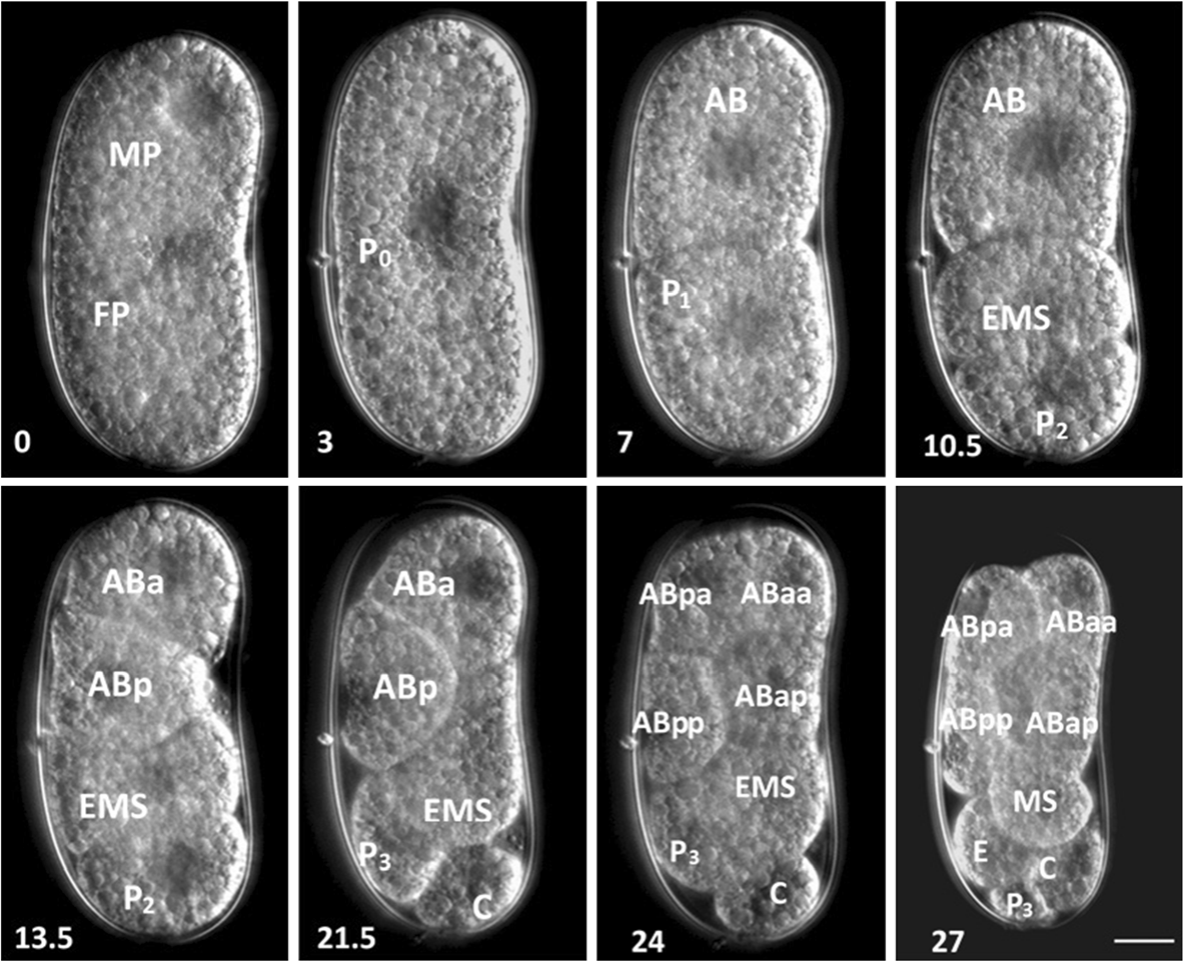
Time-lapse DIC micrographs of *H. glycines* early embryo development. Individual eggs were observed every half hour until the eight-cell stage. The time in hours (*lower left corner*) was recorded for each of the cell divisions. Cell labels are based on *Caenorhabditis elegans* and inferred based on positional homology in other nematode species [10, 14, 25, 27]. Scale, 15 μm

To better understand the hatching behavior of *H. glycines*, we first resolved the timeline of pre-hatch development using several observational techniques. Single cell eggs were rarely found. We, therefore, started most of our observations at the two-celled stage. We observed a similar sequence of pre-hatch developmental stages (2, 3, and 4-celled stages, gastrula, tadpole, J1, and J2) as recorded for other plant-parasitic nematodes [10, 14]. Furthermore, we traced the early cell lineage of some embryos. The first cell division was transverse and slightly unequal, producing the P_1_ and AB blastomeres (Fig. 1, 7 h). This asymmetry determined the anterior-posterior axis. P_1_ divided to produce EMS and P_2_ (Fig. 1, 10.5 h). Following this division, AB divided to form the ABa (a-anterior) and ABp (p-posterior) blastomeres (Fig. 1, 13.5 h). Similar to other previously described Clade 12 nematodes, we observed a linear arrangement in the four-celled embryo (ABa, ABp, EMS, and P_2_) indicative of an I_3_-Type blastomere arrangement [27]. Following the four-celled linearly arranged embryo, the P_2_ cell divided to produce C and P_3_ (Fig. 1, 21.5 h). ABa and ABp then divided to produce anterior and posterior pairs (ABaa, ABap, ABpa, ABpp) (Fig. 1, 24 h). Following the AB cell divisions, EMS divided to form MS and E, resulting in an eight-celled embryo (Fig. 1, 27 h). The cell lineage of individual animals was not traced following the eight-cell stage due to the refractive optical properties of the embryo, as described for other plant-parasitic nematodes [10].

**Fig. 2:**
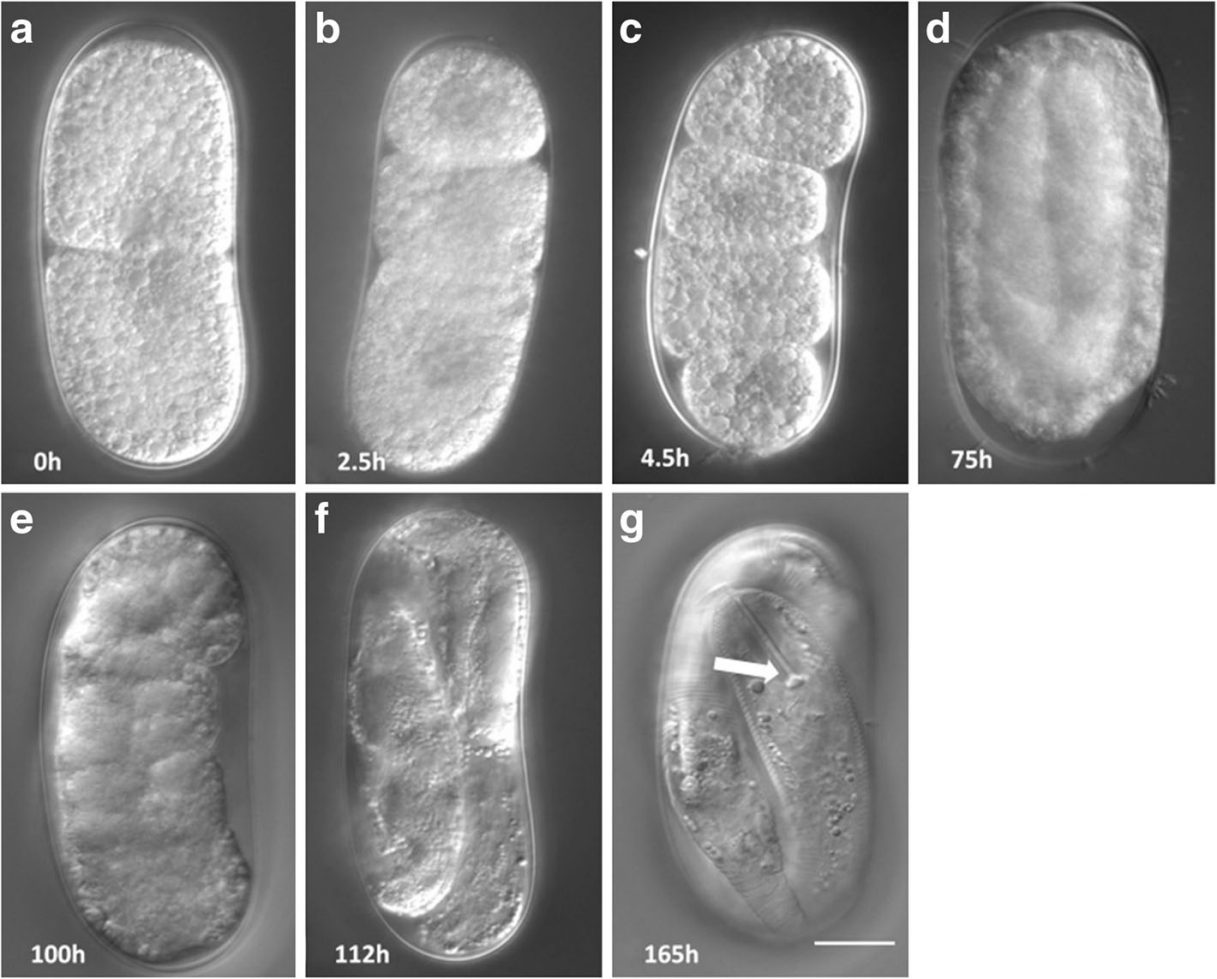
Representative DIC micrographs of selected developmental stages in *H. glycines* and their corresponding timeline in hours after the 2-cell egg. Major pre-hatch development stages were determined through long-term imaging in hanging drops. **a** 2-cell stage; **b** 3-cell stage; **c** 4-cell stage; **d** gastrula; **e** tadpole; **f** J1; **g** J2 with fully formed stylet (*arrow*). Scale, 15 μm

Following the early cell divisions, rapid longitudinal and transverse cell divisions led to recognizable developmental stages. The onset of gastrulation occurred approximately three days following the beginning of our observations (Fig. 2d). After approximately four days, the embryo began to move during the tadpole stage (Fig. 2e), and soon after formed a worm-shaped J1 (Fig. 2f). At this time the body was readily differentiated into two zones of dissimilar densities. The light zone is presumed to form the anterior end of the nematode while the dark zone is thought to develop into the intestine. A stylet was observed in J2s (Fig. 2g). The fully formed unhatched J2 moved within the egg. It kept circulating within the eggshell, with occasional bouts of quiescence, exploring locations to make a slit near a pole of the egg from which to emerge. Despite our synchronization, there was substantial variability in the developmental timeline among individual eggs within a treatment. This may be due to slight differences in environmental conditions or to genetic differences among eggs. Recently, the pre-hatch development and early cell lineage were described in the root-knot nematode *M. incognita* [14]. While *Meloidogyne* spp. and *Heterodera* spp. are both plant-parasitic species and found in the same phylogenetic clade, they display many differences in their biology and are thought to have evolved from different immediate common ancestors [28]. Interestingly, while the pattern of major pre-hatch development was similar in both species, the speed of development was much faster in *H. glycines*. For example, in *H. glycines* the E cell was produced in approximately one day at 25 °C while *M. incognita* requires almost five days at 22 °C [14].

### *H. glycines* pre-hatch development is not affected by hatching stimulants or oviposition location

**Fig. 3:**
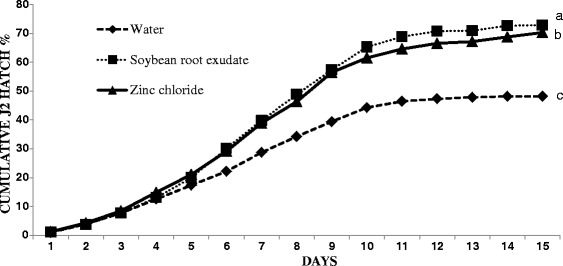
*Heterodera glycines* hatching is increased by the presence of soybean root exudate or ZnCl_2._ Asynchronous eggs were examined for hatching each day for 15 days in sterile distilled water, soybean root exudate, and ZnCl_2_. The experiment was repeated once with three replications; each replication consists of 1000 eggs. Different letters at the end of the curves indicate statistically significant differences (*α* = 0.05)

Compared with the N2 *C. elegans* laboratory strain, *H. glycines* strains are genetically variable. To confirm that our strain of *H. glycines* displays a host-mediated hatching response and to ensure the efficacy of our host exudate extraction methods, we tested the effect of different hatching stimulants on an asynchronous population of *H. glycines* eggs. As expected, the number of eggs that hatched in soybean root exudate and ZnCl_2_, a known hatching stimulant, was significantly greater than in water (*P* < 0.0001) (Fig. 3). Numerous studies have demonstrated that *H. glycines* hatching increases in the presence of a host plant or other stimulants [3, 21, 29, 30]. It is unknown whether this is due to manipulation of the rate of embryogenesis or due to stimulation once the J2 has completely developed. To test this, we examined the rate of embryogenesis in synchronized eggs exposed to different hatching stimulants. There were no obvious differences in the pre-hatch developmental timelines among eggs in water, host exudate, or ZnCl_2_ (Table 1).

**Table 1:** Hatching stimulants do not affect the pre-hatch development timeline of *Heterodera glycines*

Developmental stage	Water (n)	Zinc chloride (n)	Soybean root exudate (n)
3 cell	2.67 ± 1.15 (3)	3.24 ± 1.25 (4)	3.20 ± 1.3 (6)
4 cell	4.67 ± 2.08 (3)	5.33 ± 1.53 (3)	5.25 ± 0.96 (7)
Gastrula	75.71 ± 9.25 (6)	72.60 ± 1.51 (5)	70.60 ± 4.35 (7)
Tadpole	100.50 ± 8.31 (6)	97.25 ± 4.57 (4)	93.80 ± 5.77 (5)
J1 stage	112 ± 7.66 (7)	113.80 ± 9.49 (5)	107.72 ± 10.12 (10)
J2 stage	168.29 ± 7.16 (7)	165.20 ± 9.76 (5)	166.14 ± 10.16 (11)

Fifteen synchronized two-celled eggs were placed in water, soybean root exudate or 3 mM ZnCl_2_ and examined at variable time points. Data are means ± s.d. of hours to reach a specific developmental stage following the two-cell stage. Numbers in parentheses indicate number of animals observed at that specific stage out of the fifteen. For some animals, observation of the specific stage was missed. The J2 stage was assessed by the presence of a fully formed stylet. No statistically significant differences were found among treatments

It was also previously reported that unsynchronized *H. glycines* eggs from egg sacs hatch more rapidly than encysted eggs [8]. One possible explanation is that egg sac eggs develop faster than encysted eggs. Alternatively, egg sac eggs may be older and therefore reach the fully formed J2 stage earlier. To test these possibilities, we examined the development of synchronized eggs from each source beginning at the two-cell stage. Similar to our hatching stimulant data, we found no difference between the pre-hatch developmental timelines of encysted eggs and egg sac eggs (Table 2).

**Table 2:** Pre-hatch development timeline of *Heterodera glycines* is not affected by oviposition location

Developmental stages	Encysted eggs (n)	Egg sac eggs (n)
3 cell	2.67 ± 1.15 (3)	3.50 ± 0.84 (6)
4 cell	4.67 ± 2.08 (3)	4.71 ± 0.49 (8)
Gastrula	75.71 ± 9.25 (6)	69.86 ± 2.97 (7)
Tadpole	100.50 ± 8.31 (6)	95 ± 0 (5)
J1 stage	112 ± 7.66 (7)	126.20 ± 9.56 (10)
J2 stage	168.29 ± 7.16 (7)	172.54 ± 9.34 (10)

Fifteen synchronized two-celled eggs from the egg sac or the cyst were examined daily in water. Data are mean ± s.d. hours after the two-cell stage. Numbers in parentheses indicate number of animals observed at that specific stage out of the fifteen. For some animals, observation of the specific stage was missed. The J2 stage was assessed by the presence of a fully formed stylet. No statistically significant differences were found between treatments

### *H. glycines* stylet and ventral nerve cord continue to develop in pre-hatched J2s

**Fig. 4:**
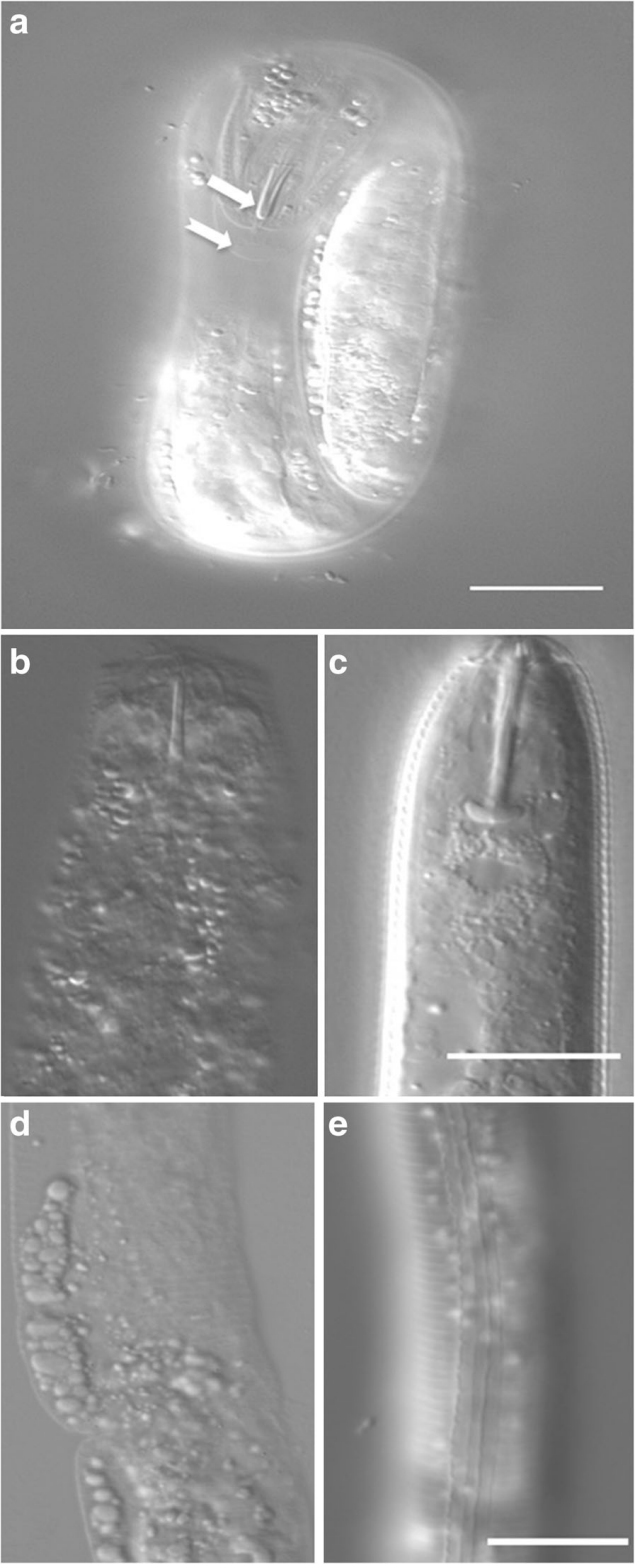
DIC micrographs demonstrating that development of the stylet and cuticle in pre-hatched J2 *Hetrodera glycines* continues beyond the J1 to J2 molt. Pre-hatch J2s **a** immediately following the J1 to J2 molt retaining its old J1 cuticle (*lower arrow*) and without a completely developed stylet (*upper arrow*). Early pre-hatched J2s mechanically removed from the eggshell (**b** and **d**) show an incomplete stylet, including a complete lack of the stylet shaft and knobs **b** and a lack of longitudinal ridges (alae) along the lateral field of the cuticle **d**. In comparison, a fully formed J2 has a complete stylet **c** and well developed alae **e**. Scale, 15 μm

**Fig. 5:**
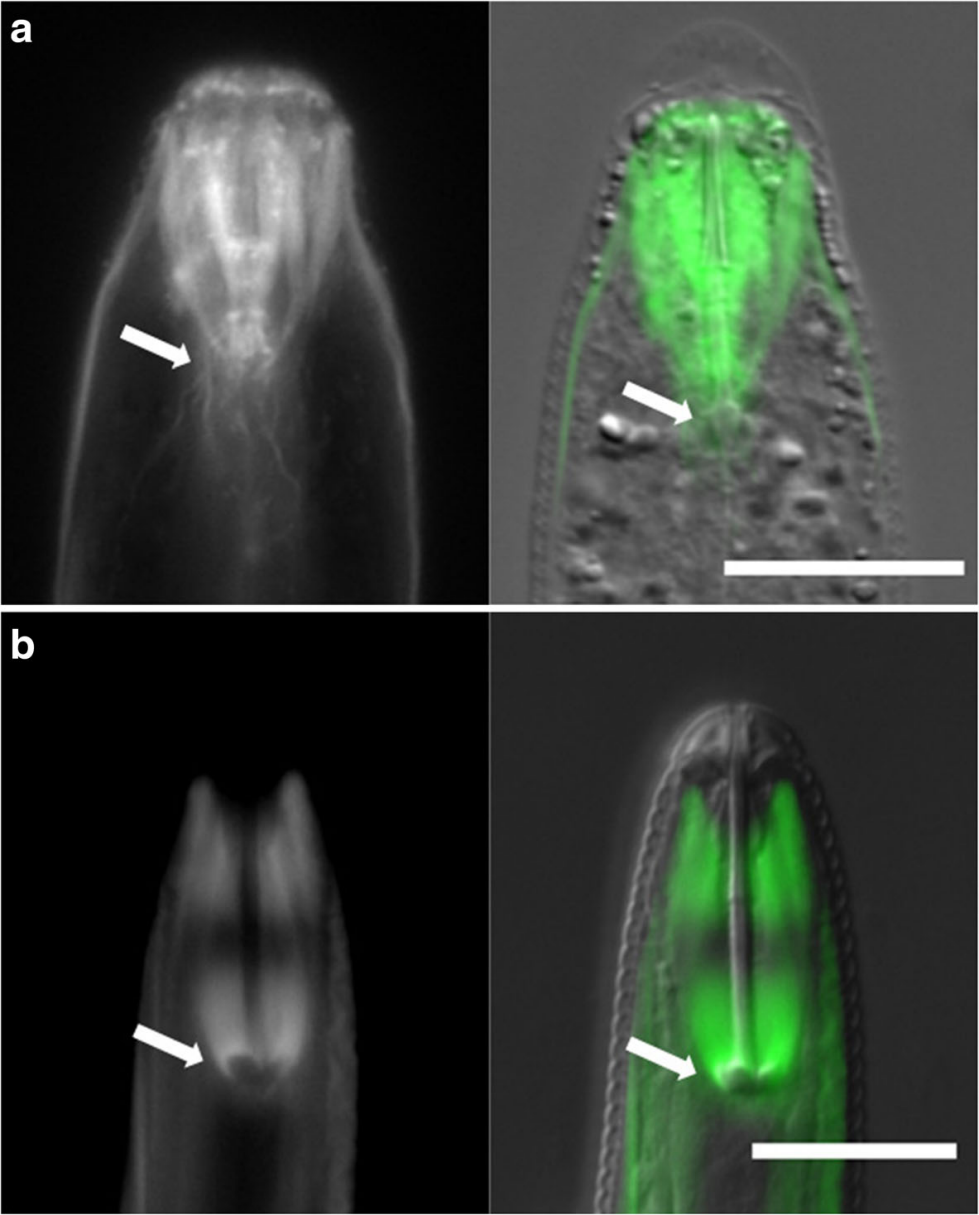
The stylet protractor muscle development precedes the complete development of the stylet. Pre-hatched **a** and hatched **b** J2 *Heterodera glycines* were stained with phalloidin and imaged using epifluorescence (*left*) and DIC optics (*overlay, right*). The stylet protractor muscles were present prior to the complete formation of the stylet knobs (*arrows*). Scale, 15 μm

Most plant-parasitic nematodes hatch following the molt from J1 to J2. During our studies, we observed that the anatomy of unhatched J2s did not appear identical to hatched J2s. Specifically, we noted pre-hatch J2s with a partially shed J1 cuticle, indicative of the first molt, but which did not have a fully developed stylet (Fig. 4a). The *H. glycines* J2 stylet is a protractible hollow spear used to cut open the eggshell during hatching and for later infection. As the pre-hatched J2s continuously moved within the egg, it was difficult to observe this structure over time. Therefore, to better understand changes during J2 pre-hatch development, we carefully opened eggs containing J2s at various time points following the first molt. Nematodes were fixed and stained with DAPI and phalloidin to label nuclei and filamentous actin, respectively, and then examined with fluorescent and DIC optics. Based on our observations, we could separate J2s into distinct groups we call “early” and “late” J2. Early J2s have only the anterior cone component of the stylet (Fig. 4b). As development progresses, the shaft becomes visible followed by the stylet knobs. The stylet knobs initially appear as a slight enlargement of the shaft. Over time the stylet knobs expand in diameter and begin to show a slight curvature similar to that seen in hatched J2s (Fig. 4c). We hypothesized that the change of curvature might be due to attachment of the stylet protractor muscles and a physical deformation of the knobs. In fully developed J2s, the contractile portion of the three stylet protractor muscle cells attach to the stylet knobs and extend as ten separate elements to the cephalic framework [31]. Interestingly, our phalloidin staining showed that the stylet protractor muscles were present before the formation of the stylet knobs, suggesting that knob curvature is independent of muscle attachment (Fig. 5). We also observed differences in the structure of the cuticle between early and late pre-hatch J2s. Early pre-hatched J2s lacked obvious ridges (alae) along the lateral field of the cuticle (Fig. 4d) as seen in hatched J2s (Fig. 4e).

**Fig. 6:**
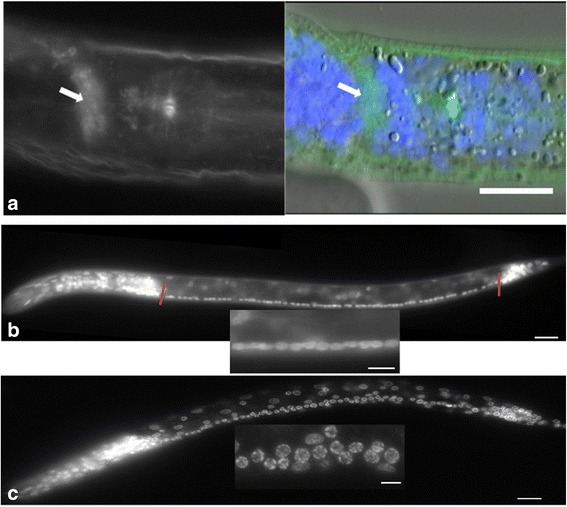
The neurodevelopment of *Heterodera glycines* early pre-hatched J2s is not complete. Pre-hatched J2 (**a** and **c**) were mechanically removed from their egg shell and stained with phalloidin and/or DAPI and compared to hatched J2s **b**. In one early pre-hatched J2 **a** we observed F-actin binding phalloidin staining (left and green in right) in the nerve ring (*arrows*). The nerve ring was identified by position and a lack of cell nuclei (blue DAPI staining in overlay right). An increase in the number of ventral nerve cord (VNC) nuclei and linearity was seen between pre-hatched **c** and hatched **b** J2s. The VNC consists of a series of motor neurons that extends from the retro-vesicular ganglion to the pre-anal ganglion (marked with red lines in B). Pre-hatched J2s had an average of 62 VNC nuclei and a less linear organization (*inset*) compared to the 65 VNC nuclei arranged in a highly linear manner in hatched J2s. A-C scale, 15 μm. Inset scale, 5 μm

We observed phalloidin staining, indicative of F-actin, in the nerve ring of one early pre-hatch J2 *H. glycines* (Fig. 6a). In *C. elegans*, actin and actin-associated proteins are often found in the nerve ring during early development [32–34]. This observation may suggest that the neurodevelopment of recently molted J2s is not complete. To test this further, we examined the ventral nerve cord of pre-hatched and hatched J2s. We found that the number of VNC neurons increased slightly from early J2s (average = 62; *n* = 20) to hatched J2s (average = 65; *n* = 20). More strikingly, the VNC of early pre-hatched J2s appeared more disorganized than in hatched J2s (Fig. 6b and c). These data support our hypothesis of continued neuronal development following the J1 to J2 molt.

## Discussion

Hatching is the result of highly regulated metabolic and developmental events that are responsive to both endogenous and environmental factors [35]. In addition to the importance of temperature on developmental rate, some animals are capable of modulating their hatching based on less obvious environmental cues [35]. Certain species of freshwater snails are capable of suppressing pre*-*hatch development based on signals received from conspecific individuals [36]. Similarly, turtles can adjust their development to synchronize hatching via embryo-embryo communication [16]. The cyst nematodes, including *H. glycines*, regulate hatching based on the presence of a host [3, 37]. The hypothesis that the complex hatching behavior of *H. glycines* is partly due to host-mediated changes to embryogenesis was previously untested.

Our results do not support the hypothesis that hatching stimulants affect *H. glycines* embryo development. We found no obvious differences in the timing of pre-hatch development of *H. glycines* when exposed to hatching stimulants. Instead, the stimulants likely influence the hatching decision once the fully formed J2 has developed. Similar to our hatching stimulant data, we found no obvious differences in the timing of pre-hatch development between egg sac eggs and encysted eggs. Thompson and Tylka [8] found that asynchronous egg sac eggs hatch sooner than encysted eggs. They suggested that rapid hatching of egg sac eggs compared to encysted eggs may be due to the gelatinous egg sac containing more mature eggs [8]. Anecdotally, we also observed a greater proportion of mature eggs in the egg sac than in cysts. One caveat to ours and other studies is that manipulation of eggs within the lab may lead to artifacts in development and behavior not seen in nature. An additional question that remains to be addressed is the mechanism and types of quiescence that occur in pre-hatched J2s. Approximately 30% of eggs were unresponsive to hatching stimulants (Fig. 3). This may suggest these eggs are in a state of long-term dormancy distinct from that found in eggs which respond to stimulation [38].

Our data show that while the pattern of embryogenesis is very similar to most previously described plant-parasitic nematodes, the rate of development is variable. Previous research has demonstrated variability in early embryogenesis among diverse nematode species [10, 27, 39]. *C. elegans* embryos develop to the eight-cell stage in less than one hour [25]. Although the cleavage pattern of early embryogenesis varies among nematodes, we found *H. glycines* has a very similar cleavage pattern to *M. incognita* [14]. However, the pre-hatch development of *H. glycines* was substantially faster than *M. incognita* [14]. We found that *H. glycines* developed from a one-cell egg to eight cells in approximately one day, and hatched after eight days. *M. incognita* took approximately five days to form the eight-cell embryo and hatched after 21 days [14]. This difference in timing may be due to the incubation temperature. While we maintained our eggs at 25 °C, Calderón-Urrea et al. [14] incubated their *M. incognita* eggs at 22 °C. Interestingly, an earlier study suggested that *M. incognita* formed J2s within seven days at 20 °C [40]. Unlike the laboratory maintained N2 strain of *C. elegans*, laboratory-maintained populations of plant-parasitic nematodes are established from diverse locations and likely exhibit genetic and phenotypic diversity. It will be interesting to directly examine the variability in embryogenesis and hatching among diverse populations of *H. glycines*.

A protractible stylet mouthpart is an adaptation found in all plant-parasitic nematodes. The stylet is used for infection and, in some species, for hatching. The mechanisms regulating the development of this important structure are unknown. Unlike *C. elegans*, most plant-parasitic nematodes undergo the first molt within the egg and hatch as J2s. We observed the developing stylet cone during the first molt of *H. glycines*. Our observations are similar to those previously described in the plant-parasitic nematode *Hoplolaimus columbus* [41]. Furthermore, we found that individual structures like the stylet and stylet protractor muscles continued to develop in pre-hatch J2s. While we did not specifically test if hatching stimulants affect the progression of development during this stage, our analysis suggested there were no differences among different hatching stimulants in the time to reach either the J1 or the J2 (Table 1).

The stylet protractor muscles were present in early pre-hatch J2s before the formation of stylet knobs. In *C. elegans*, esophageal muscles are thought to secrete the cuticle lining the stoma lumen [42]. It is tempting to speculate that *H. glycines* stylet protractor muscles secrete the cuticle that forms the stylet. However, Endo [43] suggested that the cuticle-based stylet is secreted by arcade epithelial cells. It may be possible to ablate individual stylet protractor muscle cells to test these hypotheses. In addition to our finding of post-molt stylet development, we also uncovered changes to the nervous system. It is uncertain from our data whether these changes to the nervous system are required for hatching behavior. Further investigation into pre-hatch development may lead to strategies for controlling this parasite.

## Conclusions

Embryogenesis in the parasitic nematode *H. glycines* is decoupled from hatching stimulants or oviposition location. While the pattern of early embryonic development in *H. glycines* was very similar to the closely related parasitic nematode *M. incognita*, the rate of *H. glycines* development was substantially faster. The continued development of the stylet, nerve ring, and the ventral nerve cord in pre-hatched J2s may suggest they are required structures for *H. glycines* hatching.

## Abbreviations

DAPI: 4’, 6-diamidino-2-phenylindole
VNC: Ventral nerve cord
RVG: Retro-vesicular ganglion
PAG: Pre-anal ganglion.
